# Tailoring Artificial Solid Electrolyte Interphase via MoS_2_ Sacrificial Thin Film for Li-Free All-Solid-State Batteries

**DOI:** 10.1007/s40820-025-01729-w

**Published:** 2025-04-18

**Authors:** Dong-Bum Seo, Dohun Kim, Mee-Ree Kim, Jimin Kwon, Hyeong Jun Kook, Saewon Kang, Soonmin Yim, Sun Sook Lee, Dong Ok Shin, Ki-Seok An, Sangbaek Park

**Affiliations:** 1 Thin Film Materials Research Center, Korea Research Institute of Chemical Technology (KRICT), 141 Gajeong-ro, Yuseong-gu, Daejeon, 34114 Republic of Korea; 2 Department of Materials Science and Engineering, Chungnam National University, Daejeon, 34134 Republic of Korea; 3 Intelligent Sensors Research Section, Electronics and Telecommunications Research Institute (ETRI), Daejeon, 34129 Republic of Korea

**Keywords:** Molybdenum disulfide, Anode-free all-solid-state battery, Li-free all-solid-state battery, Mo metal, Lithium sulfide

## Abstract

Anode-free all-solid-state batteries (AFASSBs) are potential candidates for next-generation electric mobility devices that offer superior energy density and stability by eliminating Li from the anode. However, despite its potential to stabilize the interface between sulfide solid electrolytes (SEs) and anode-free current collectors (CCs) efficiently, a controllable approach to incorporating MoS_2_ into AFASSBs has not yet been found. Herein, we propose a strategy for stabilizing the interface of Li-free all-solid-state batteries using controllable MoS_2_ sacrificial thin films. MoS_2_ was controllably grown on CCs by metal–organic chemical vapor deposition, and the MoS_2_ sacrificial layer in contact with the SEs formed an interlayer composed of Mo metal and Li_2_S through a conversion reaction. In the AFASSBs with MoS_2_, Mo significantly reduces the nucleation overpotential of Li, which results in uniform Li plating. In addition, MoS_2_-based Li_2_S facilitates the formation of a uniform and robust SE interface, thereby enhancing the stability of AFASSBs. Based on these advantages, cells fabricated with MoS_2_ exhibited better performance as both asymmetrical and full cells with LiNi_0.6_Co_0.2_Mn_0.2_O_2_ cathodes than did cells without MoS_2_. Moreover, the cell performance was affected by the MoS_2_ size, and full cells having an optimal MoS_2_ thickness demonstrated a 1.18-fold increase in the initial discharge capacity and a sevenfold improvement in capacity retention relative to SUS CCs. This study offers a promising path for exploiting the full potential of MoS_2_ for interface stabilization and efficient AFASSB applications.

**Supplementary Information:**

The online version contains supplementary material available at 10.1007/s40820-025-01729-w.

## Highlights

- We present the first-ever fabrication of the anode-free all-solid-state battery (AFASSB) structure using a MoS_2_ sacrificial layer.
- The addition of an MoS_2_ sacrificial layer to AFASSBs could decrease the nucleation overpotential of Li and enable favorable Li formation at the interface owing to the formation of an interlayer comprising Li_2_S and Mo metal.
- The AFASSB full cell assembled with LiNi_0.6_Co_0.2_Mn_0.2_O_2_ cathodes operated successfully, demonstrating superior cycling stability and enhanced capacity relative to the cells with SUS.

**Supplementary Information:**

The online version contains supplementary material available at 10.1007/s40820-025-01729-w.

## Graphical abstract

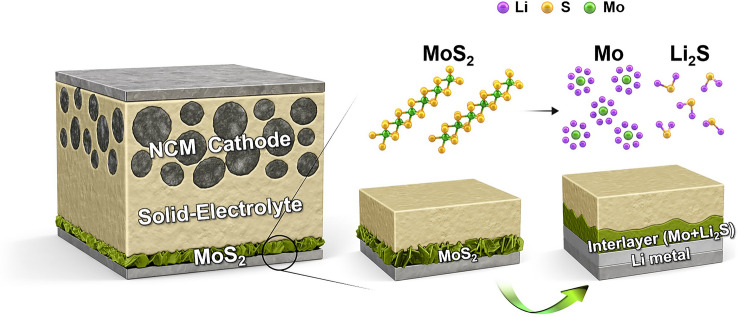

## Introduction

Despite significant research to address the demands of next-generation lithium-ion batteries, conventional lithium-ion batteries continue to face safety challenges, and their low energy density makes it difficult to meet the needs of future electronic devices [1–3]. All-solid-state batteries (ASSBs) are considered next-generation batteries where flammable liquid electrolytes are replaced with solid-state electrolytes to enhance safety and potentially increase energy density [4–8]. Among the various solid electrolytes (SEs) used in ASSBs, sulfide SEs are the most suitable because of their soft mechanical properties and Li-ion conductivity, which are comparable to those of conventional liquid electrolytes. In particular, the ductility of sulfide SEs allows for the fabrication of electrolyte and electrode layers by a simple cold-pressing process to improve processability and enable thin-film preparation and large-scale production [9–13]. Furthermore, lithium (Li) metal, with its high theoretical capacity (3,860 mAh g^−1^), is a suitable anode material for sulfide-based ASSBs that target high energy density, which makes it a promising alternative to the graphite (theoretical capacity of 372 mAh g^−1^) that is widely used in current Li-ion batteries [11, 14–17]. Nevertheless, the performance of cells having Li metal anodes is unsatisfactory because most of the excess Li metal does not react during cycling. To overcome these limitations, anode-free lithium-metal batteries (AFLMBs) have recently received attention owing to their high energy density without containing excess lithium [18–21]. In AFLMBs, the cathode is the sole source of Li during charging, which eliminates the need to handle Li metal foils during cell assembly, thereby simplifying fabrication and reducing costs [11, 22]. Despite the potential of AFLMBs, they still exhibit low coulombic efficiency and poor cycling performance owing to the reaction between Li metal and the electrolyte, as well as the nonuniform formation of lithium metal on the current collector (CC) [23, 24]. Hence, proposing various improvement strategies, such as regulating the uniform deposition of lithium ions and constructing high-quality protective layers, is crucial for enhancing the performance of AFLMBs.

In various studies on AFLMBs or lithium-metal batteries, the modulation of the CCs, such as coating functional layers onto the CCs and modifying the solid electrolyte interface (SEI) passivation layer, has been proposed to improve the uniformity of the initial lithium deposition and the stability of batteries [20, 25]. Since the Samsung Group reported that Ag nanoparticles in Ag-C composite layers provide Li nucleation sites and promote uniform Li plating onto CCs in ASSBs [11], several studies have been conducted to improve the uniformity of Li deposition by coating various Li-friendly metals onto CCs as functional layers [26–28]. Park et al. demonstrated a 3D architecture that was tailored for Li-free ASSBs by modifying the surface of a porous Ni CC with a functionalized Ag-C layer [29]. Based on comparative studies of various lithiophilic metals, the Choi and Lee groups respectively reported Mg to be a promising functional layer for AFLMBs or Li-free ASSBs because of its low reaction barrier with Li, which facilitates reversible Li plating and stripping [26, 27]. Lee et al. demonstrated that a combination of Ag and In in AFLMBs exhibited superior performance relative to other candidate metals, and this was attributed to the beneficial properties of the Ag-In alloy, including its high Li adsorption energy and the formation of a chemically compatible interface with the sulfide SEs [28]. Gu et al. demonstrated that modulating the surface morphology of the CCs is crucial to enhancing the reaction sites between the SEs and CCs in AFLMBs [30]. However, previously reported lithiophilic metals and their composites have limitations in industrial applications owing to factors such as high material costs, the fire-related instability of some materials, and their relatively thick layers. In addition, a comprehensive approach that integrates the introduction of functional layers with modifications to surface morphology for the effective operation of anode-free all-solid-state batteries (AFASSBs) has yet to be reported.

Transition metal dichalcogenides (TMDs) have attracted attention in recent studies owing to their earth abundance, low cost, exceptional electronic properties, and energy-harvesting performance [31–35]. MoS_2_, a typical TMD, is considered to be an ideal material for constructing high-quality Li-protective layers due to its several advantageous properties [36, 37]. Recent studies have revealed that MoS_2_ is an excellent Li^+^ conductor that is capable of reacting with lithium, and that using MoS_2_ as a protective layer for Li metal can effectively establish a protective barrier between the Li metal and the electrolyte, thereby enhancing the battery’s cycle stability [38–41]. Choi et al. reported that MoS_2_ could react with Li^+^ ions to form an intermediate layer of Li_2_S and Mo metal, thereby enhancing the wettability of Li and improving its interface [42]. Wang et al. demonstrated that MoS_2_ could enhance battery capacity because of the ability of Mo metal to accommodate a large amount of Li^+^ [43]. Despite the promising properties of MoS_2_ materials, systematic research on the application and optimization of these materials in lithium-metal batteries with SEs remains insufficient. Furthermore, the effective operation of MoS_2_-based AFASSB full cells has not been implemented, despite their potential for application in AFASSBs and ASSBs.

In this study, we report an approach for stabilizing the AFASSB interface using a controllable MoS_2_ sacrificial layer. Generally, pristine steel use stainless (SUS) exhibits low reactivity with sulfides, which makes it a potential replacement for copper as the CC in AFASSBs [11]. However, the structural incompatibility of SUS with lithium and the inadequate contact between the SEs and CCs may lead to uneven lithium-ion flux [44, 45]. To address this issue, we propose a strategy for introducing MoS_2_ sacrificial thin films to form an interlayer of Mo metal and Li_2_S on the SUS CCs. The MoS_2_ were grown on CCs by metal–organic chemical vapor deposition (MOCVD), and MoS_2_ morphology was modulated architecture vertically standing nanosheets on CCs. Vertical MoS_2_ nanosheets have a large specific surface area and active sites [46, 47], which can increase their contact area with the SEs and promote the formation of Mo metal and Li_2_S for uniform initial Li plating. By performing a systematic and integrated electrochemical analysis of the asymmetric and full cells, we demonstrated that the addition of a MoS_2_ sacrificial layer to AFASSBs could decrease the nucleation overpotential of Li and enable favorable Li formation at the interface owing to the formation of an interlayer comprising Li_2_S and Mo metal. Thus, the cycling stability of the MoS_2_-based asymmetric cells was significantly improved by more than 3.2-fold relative to that of the bare SUS cells, and the cell properties were affected by the size of MoS_2_. Moreover, the AFASSB full cell assembled with LiNi_0.6_Co_0.2_Mn_0.2_O_2_ (NCM 622) cathodes operated successfully, demonstrating superior cycling stability and enhanced capacity. This study proposes a facile and efficient strategy for exploiting the full potential of MoS_2_ for practical AFASSB applications.

## Experimental Section

### Preparation of MoS_2_ for Current Collectors

MoS_2_ was directly grown on SUS substrates for various times (3, 15, and 45 min) in a metal–organic chemical vapor deposition (MOCVD) system with Mo(CO)_6_ and H_2_S gases as the Mo and S precursors, respectively. Mo(CO)_6_ was vaporized and delivered to a quartz tube using Ar gas at 50 standard cubic centimeters per minute (SCCM). The flow rate of H_2_S gas was 150 SSCM. The growth pressure and temperature were fixed at 2 Torr and 260 °C, respectively.

### Synthesis of Solid Electrolyte and Modified Cathode

Li_6_PS_5_Cl was prepared as the SE using a typical planetary milling method. The raw materials were Li_2_S (> 99.9%, Sigma-Aldrich), P_2_S_5_ (> 99.9%, Sigma-Aldrich), and LiCl (> 99.9%, Sigma-Aldrich), and these were placed, at an appropriate molar ratio, into ZrO_2_ ball-mill jars containing Ø3 mm ZrO_2_ balls, where the ball-to-powder weight ratio was 25:1. All the weight-ratio calculations and input procedures were carried out in an Ar-filled glove box. The powders were mechanically mixed using planetary ball milling at 200 rpm for 30 min. Subsequently, the mixture was milled at 650 rpm for 18 h. Then, the synthesized Li_6_PS_5_Cl was heated at 500 °C for 2 h in quartz tubes. In addition, LiNbO_3_-coated LiNi_0.6_Co_0.2_Mn_0.2_O_2_ (NCM 622) was prepared as an active cathode composite layer by vacuum distillation and sintering. First, C_4_H_4_NNbO_9_∙nH_2_O and C_2_H_3_O_2_Li∙2H_2_O were dissolved in ethanol, and NCM 622 was evenly dispersed in ethanol in a rotary evaporation flask. Afterward, a mixed solution containing C_4_H_4_NNbO_9_∙nH_2_O and C_2_H_3_O_2_Li∙2H_2_O was slowly dropped into the NCM 622 solution, and an intermediate material was obtained by vacuum distillation at room temperature (RT). Finally, the LiNbO_3_-modified NCM 622 was obtained by sintering at 350 °C in an O_2_ atmosphere.

### Material Characterization

**Fig. 1:**
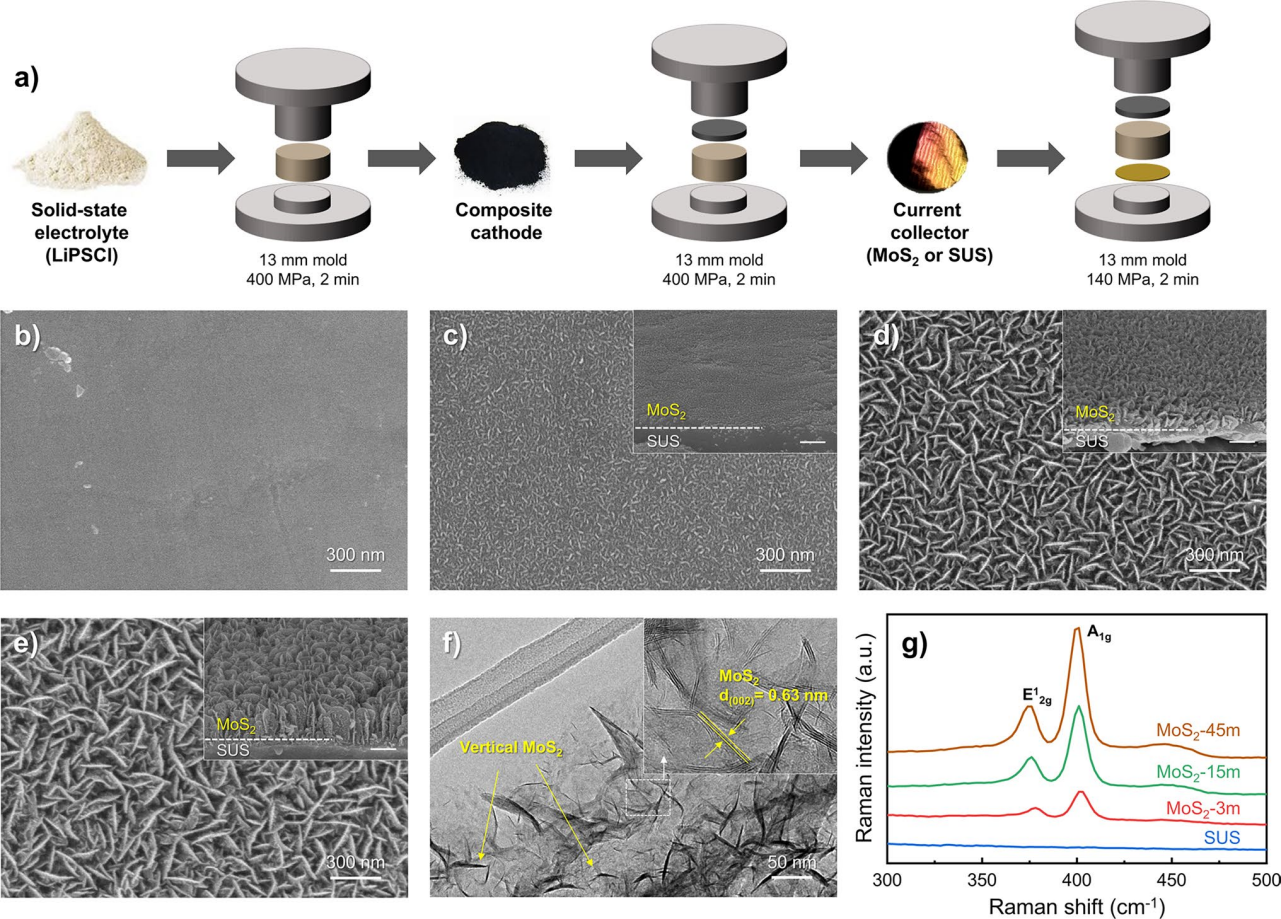
**a** Schematic fabrication process of the AFASSBs with anode-less CCs. Top- and cross-view SEM images of **b** SUS CCs and MoS_2_ on CCs with various growth times: **c** MoS_2_-3m at 3 min, **d** MoS_2_-15m at 15 min, and **e** MoS_2_-45m at 45 min. **f** Low-magnification TEM image of MoS_2_-15 m and HRTEM image (inset). **g** Raman spectra of various samples (SUS CCs, MoS_2_-3 m, MoS_2_-15 m, and MoS_2_-45 m)

The sample morphology was investigated using scanning electron microscopy (SEM, Hitachi S-4800) and high-resolution transmission electron microscopy (HRTEM; JEM-ARM200F, JEOL). The microstructural properties of the samples were investigated via micro-Raman spectroscopy at an excitation wavelength of 532 nm using a charge-coupled device detector (UniThink Inc., UR1207J). The crystallinity of the samples was evaluated by X-ray diffraction (XRD, Bruker D8 Discover, Bruker-AXS). The chemical states and composition of the samples were characterized using X-ray photoelectron spectroscopy (XPS; Thermo VG Scientific) with an Al Kα radiation source. For asymmetric cells using SEs, 300 mg of argyrodite Li_6_PS_5_Cl as the SEs layer was placed into a polyether ether ketone (PEEK) mold with a diameter of 13 mm and compressed at 400 MPa for 2 min. The CCs were placed on one side of the SE pellet and pressed at 140 MPa. The In-Li alloy based on the Li foil (as the counter electrode) was then laid on the other side of the pellet and pressed at 140 MPa. For the full cells, as shown in Fig. 1a, the SE pellet was prepared in the same manner as for the asymmetric cells, and 34 mg of the composite cathode was loaded onto one side of the SE pellet and pressed at 400 MPa. Subsequently, the bare or MoS_2_-containing CCs were placed on the opposite side of the pellet and compressed at 140 MPa. The composite cathode was composed of LiNbO_3_-modified NCM 622 (as the active material), SEs, and vapor-grown carbon fiber (VGCF) (as the conductive agent) at a weight ratio of 65:30:5. The SEs and VGCF were first mixed, and the active material was then added to the mixture and mixed in the same manner. All electrochemical measurements were conducted at 60 °C with normal stack pressure.

## Results and Discussion

### Cell Fabrication Design and Structural Characterizations of Materials

Figure 1a shows the fabrication process for the AFASSBs with anode-free CCs. To limit the experimental variables to the CCs on the anode side, we first confirmed the correct synthesis of the argyrodite (Li_6_PS_5_Cl) corresponding to the sulfide SEs. Figure S1a shows the X-ray diffraction (XRD) pattern of the Li_6_PS_5_Cl electrolyte. The XRD pattern indicates that the Li_6_PS_5_Cl is present in the well-formed argyrodite *F*-43* m* structure [11], with a broad peak at approximately 19° corresponding to Kapton tape [48]. Figure S1b shows the Nyquist plots and fitting results for the Li_6_PS_5_Cl electrolyte at room temperature (RT). The total impedance and lithium-ion conductivity of the Li_6_PS_5_Cl, as calculated from the electrochemical impedance spectroscopy (EIS) Nyquist plot, are approximately 336.44 Ω and 1.8 mS cm^−1^ at RT, respectively (Table S1). Typical argyrodites are reported to have an ionic conductivity (> 1 mS cm^−1^) that is suitable for ASSBs [11, 49], indicating that the Li_6_PS_5_Cl prepared in this study is an appropriate SE for ASSBs or AFASSBs. Figure 1b–e shows the morphological characteristics of SUS CCs and MoS_2_ on CCs for various MoS_2_ growth times (3, 15, and 45 min; herein referred to as MoS_2_-3m, MoS_2_-15m, and MoS_2_-45m, respectively); the inset shows a cross-view scanning electron microscopy (SEM) image. As shown in Fig. 1b, c, the pristine SUS CCs exhibited a flat surface without sheets, whereas the MoS_2_ nanosheets covered the surface of the CCs in the 3-min condition (MoS_2_-3m). When the growth time was increased to 15 and 45 min, the MoS_2_ nanosheets continued to grow, and the nanosheet sizes of MoS_2_-15m and MoS_2_-45m reached approximately 180 and 450 nm, respectively, as illustrated in Fig. 1d, e. Sufficient S^2–^ conditions in the MOCVD reaction can promote the vertically aligned MoS_2_ nanosheets on CCs, thereby improving the accessible surface area [50, 51]. Transmission electron microscopy (TEM) and Raman analyses were conducted to investigate the microstructural properties of MoS_2_. Figure 1f shows the low-magnification TEM image of MoS_2_-15 m, which reveals that the vertical MoS_2_ nanosheets are uniformly distributed. Moreover, a high-resolution TEM image, obtained from the white-boxed region in Fig. 1f, shows that the MoS_2_ nanosheets have a lattice spacing of approximately 0.63 nm, corresponding to the (002) plane of MoS_2_ (see inset of Fig. 1f) [52]. Figure 1g shows the Raman spectrum for each of the various samples. Whereas the SUS exhibited no peaks, the Raman spectra of all MoS_2_ samples displayed the characteristic E^1^_2g_ and A_1g_ modes of MoS_2_. The E^1^_2g_ mode is attributed to the in-plane vibrations of the Mo and S atoms, whereas the A_1g_ mode is due to the out-of-plane vibrations of the S atoms [53].

### Electrochemical Properties and Structural Characterizations: Effect of MoS_2_

**Fig. 2:**
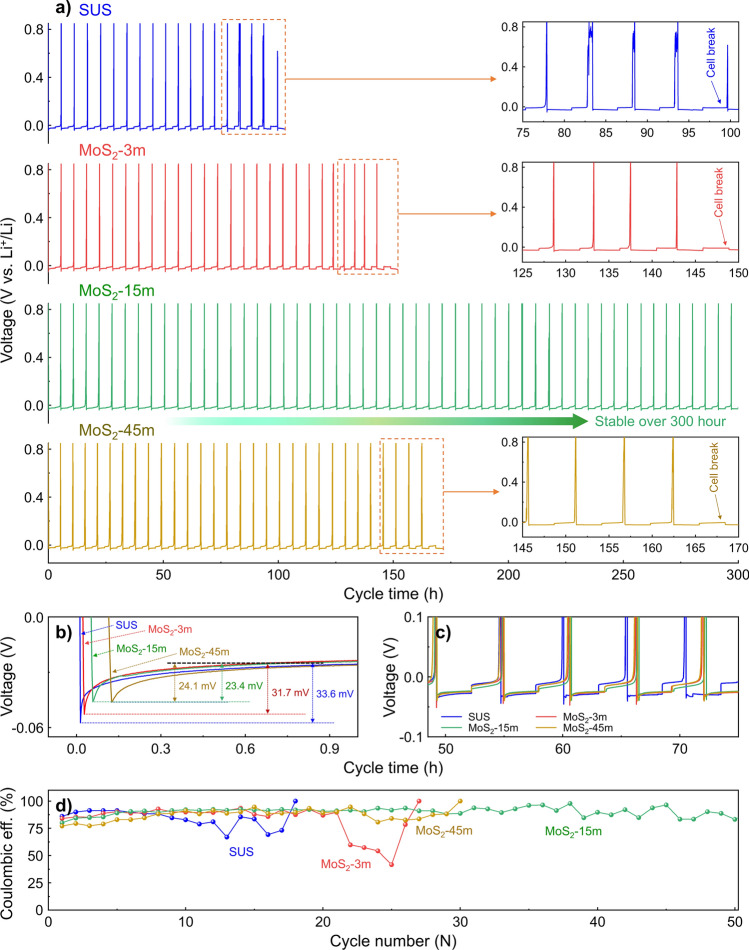
**a** Asymmetric half-cell performances with various samples (SUS CCs, MoS_2_-3m, MoS_2_-15m, and MoS_2_-45m). **b** Voltage profiles for each sample during initial Li plating. **c** Detailed magnified asymmetric cell test curves for SUS CCs, MoS_2_-3m, MoS_2_-15m, and MoS_2_-45m at cycle medium times (approximately 48–75 h). **d** Coulombic efficiency of the asymmetric cells as a function of cycle number

An asymmetric half-cell test using an In-Li alloy was carried out to assess the performance of both the pristine SUS CCs and MoS_2_-coated CCs, thereby demonstrating our research strategy. The half-cell tests involved plating at a current density of 0.3 mA cm^−2^ for 3 h, followed by stripping at the same current density with a cutoff voltage of 0.3 V. Figure 2a exhibits the Li plating/stripping cycling of various asymmetric half-cells (SUS CCs, MoS_2_-3 m, MoS_2_-15 m, and MoS_2_-45 m). As shown in Fig. 2a, the pristine SUS CCs demonstrated a relatively short cycle life, with a short circuit occurring after 95 h of cycling. In contrast, the asymmetric cell with the MoS_2_-coated CCs demonstrated improved galvanostatic cycling stability, indicating that the addition of MoS_2_ facilitated kinetic Li plating and stripping. Comparing the stability of various MoS_2_ samples, MoS_2_-15 m demonstrated stable operation and the longest cycle life, which surpassed 300 h, followed by MoS_2_-45 m at 165 h and MoS_2_-3 m at 145 h. To further validate the performance enhancement induced by the addition of MoS_2_, we also conducted critical current density (CCD) analysis and galvanostatic cycling tests at higher current densities. The CCD of MoS_2_-15 m was higher than that of SUS CC, consistent with the galvanostatic cycling results (Fig. S2a, b). Furthermore, MoS_2_-15 m exhibited superior stability (> 100 h) compared to conventional SUS CC in galvanostatic cycling tests conducted at a current density of 0.5 mA cm^−2^ with an areal capacity of 1.5 mAh cm^−2^ (Fig. S2c). This enhancement can be attributed to improved lithium plating/stripping characteristics, which promote uniform lithium deposition and facilitate a more even distribution of the applied current over a larger area [54]. The voltage profiles of the asymmetric cells in Fig. 2b show the nucleation overpotentials of the SUS CCs, MoS_2_-3 m, MoS_2_-15 m, and MoS_2_-45 m. The nucleation overvoltage of Li is defined as the gap between the bottom of the voltage dip and the flat region of the voltage plateau within the voltage profile, and the overpotential is correlated with the uniform plating of Li [55]. The SUS CCs exhibited a high nucleation overpotential of 33.6 mV, whereas the MoS_2_-coated CCs showed a reduced nucleation overpotential regardless of the MoS_2_ growth conditions (MoS_2_-3 m: 31.7 mV; MoS_2_-15 m: 23.4 mV; MoS_2_-45 m: 24.1 mV, respectively). The nucleation overpotential represents the additional potential required to open the SE-CC interface and promote Li nucleation and growth [56]. Thus, the reduction in the overpotential indicates that lithium nucleation and growth become easier and more uniform, which can be attributed to the introduction of MoS_2_-based materials that act as nucleation promoters to facilitate the uniform deposition of Li^+^ ions [36, 40]. In the same context, the uniformity of Li deposits in the early cycles greatly affects that of the Li deposits in subsequent cycles, and thus the stability in the entire cycle, which is consistent with the cycling tendency of our asymmetric cell. Meanwhile, although the nucleation overpotentials between MoS_2_-15 m and MoS_2_-45 m are similar, MoS_2_-45 m exhibited relatively poor cycling stability compared to MoS_2_-15 m. This difference in cycling performance based on MoS_2_ thickness may be related to the formation of an interlayer due to the conversion reaction of MoS_2_ in contact with the SE interface, which will be discussed in more detail in the following sections. Figure 2c shows a comparison of the voltage hysteresis between the four asymmetric cells during the middle cycle (approximately 48–75 h). From the intermediate cycles onward, the voltage hysteresis of the MoS_2_-coated CCs (SUS CCs, MoS_2_-3 m, MoS_2_-15 m, and MoS_2_-45 m) remains largely unchanged from its initial state, whereas the graph profile of the SUS CCs displays a gradual trend of degradation. This tendency is similarly reflected in the coulombic efficiency, which is a critical indicator for assessing the reversibility of lithium plating and stripping in asymmetric cells. Figure 2d shows the coulombic efficiency (stripping charge/plating charge) plots for various samples as a function of the cycle number. The coulombic efficiency was quantified following the protocols established in previous studies [29, 57] to evaluate the efficiency of Li formation and utilization on the surface of the CCs in the AFASSB system. The asymmetric cell with MoS_2_-coated CCs demonstrated a significantly enhanced coulombic efficiency and cycling stability. This improvement clearly suggests that the incorporation of MoS_2_ into the CCs effectively enhanced the efficiency and stability of Li plating and stripping in the AFASSB half-cell system. In addition, the average coulombic efficiency of the asymmetric cell using the MoS_2_-15 m sample (over 90%) was higher than those of the other half-cells (SUS: ~ 83.6%; MoS_2_-3 m: ~ 85.1%; MoS_2_-45 m: ~ 86.7%), which implies the presence of an optimal MoS_2_ size for efficient lithium deposition.

**Fig. 3:**
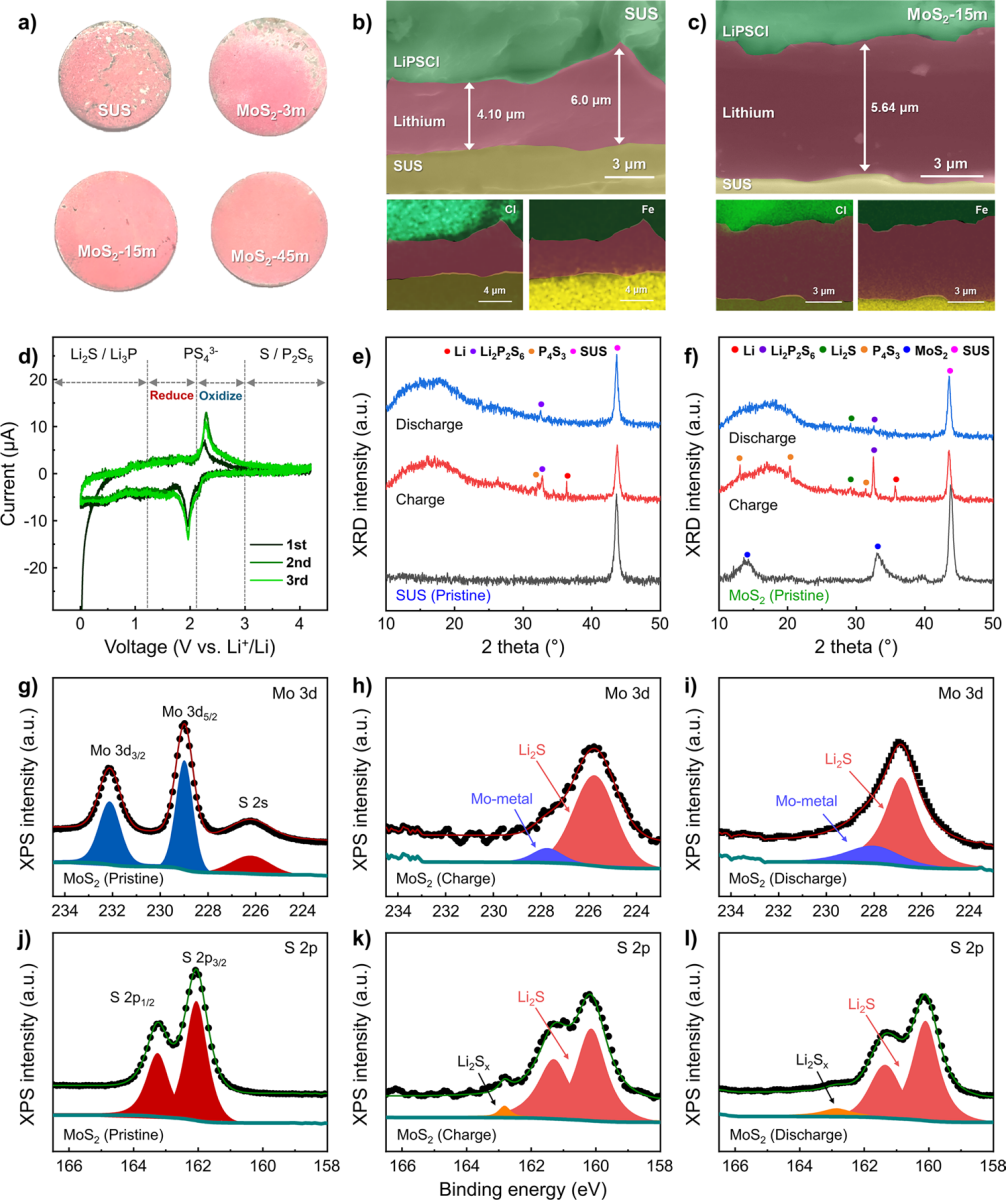
**a** Pressure paper showing the distribution of pressure over the cell area when various CCs (SUS, MoS_2_-3m, MoS_2_-15m, and MoS_2_-45m) are used. Cross-sectional SEM images and EDS mapping results for the **b** SUS and **c** MoS_2_-15m-based AFASSBs after initial Li deposition. **d** CV curve of the assembled cell based on SEs containing MoS_2_-15m. XRD results of **e** SUS and **f** MoS_2_-15m (pristine state, after lithium plating, and after lithium stripping). Deconvoluted XPS core-level spectra for MoS_2_-15m: (**g, h, i**) Mo 3*d* and (**j, k, l**) S 2*p* in pristine state, after lithium plating, and after lithium stripping, respectively

To investigate the effect of CC modification on the Li deposition behavior, we characterized the surface and interfacial morphology of the anode side of the AFASSBs using pressure-sensitive paper and SEM. Figure 3a shows the pressure distribution across the entire CC area of the various samples (SUS CCs, MoS_2_-3 m, MoS_2_-15 m, and MoS_2_-45 m) captured using a pressure-sensitive paper placed between the CC and SE layers. All the MoS_2_-coated CCs demonstrated a more even pressure distribution across the cell area than did the SUS CCs, likely owing to the increased contact area between the SEs and the CCs due to the high surface area of the vertical MoS_2_ nanosheets. Furthermore, the MoS_2_-15 m and MoS_2_-45 m samples exhibited more uniform pressure distributions than did the MoS_2_-3 m. This uniform distribution may contribute to the even dispersion of Li metal during deposition on the CCs [54], aligning with the trends observed for the half-cell results. Figure 3b, c shows the cross-sectional SEM images and energy-dispersive X-ray spectroscopy (EDS) mapping of the pristine SUS- and MoS_2_-15 m-based AFASSBs, respectively, after the initial Li deposition. A Li deposition layer is observed between the anode-side CCs and SEs in all the samples, and the presence of a Li layer was further confirmed by EDS mapping. Although the elemental Li in the Li deposition layer is not identified by EDS, the positions of the anode-side CCs and SEs are clearly distinguished in the elemental mapping of Fe and Cl, respectively. Owing to the absence of other components, the dark space between the anode-side CCs and the SEs corresponds to the Li metal layer [58]. The cell based on SUS CCs exhibited an ununiform Li thickness ranging from 4.1 to 6.0 μm, whereas the MoS_2_-15 m-based cell demonstrated a more uniform Li thickness, averaging around 5.6 μm. Additionally, the Li deposition thickness observed in the MoS_2_-15 m-based cell was comparable to the calculated Li thickness (~ 5.82 μm), derived from the Li plating areal capacity under the given cell operating conditions. This finding further confirms that the introduction of MoS_2_ promotes uniform lithium deposition, minimizing lithium loss compared to SUS. The uniform lithium distribution in the MoS_2_-15 m-based cell can be attributed to the improved contact area between the SE and the CC, resulting from a more uniform pressure distribution. Poor SE-CC contact causes localized electron and lithium transport, leading to nonuniform lithium deposition and dendrite formation [59]. In contrast, the enhanced contact area facilitated by the introduction of MoS_2_ (Fig. 3a) promotes uniform lithium distribution during deposition on the CC, ultimately enhancing the overall performance of AFASSBs. Cyclic voltammetry (CV) measurements were taken to examine the initial interfacial reactions between the SEs and the MoS_2_ CCs. Figure S2 shows the CV curves obtained using a liquid electrolyte with SUS and MoS_2_-15 m. The CV curve of the SUS current collector exhibits three pairs of redox peaks (Fig. S2a). The pair of peaks at 0 V corresponds to the Li⁺/Li redox couple, whereas the pair near 1 V arises from the Fe alloying reaction. The third pair of peaks at 2 V is assigned to the Fe^2^⁺/Fe^3^⁺ redox couple [29]. The CV curve of the MoS_2_ current collector exhibits reduction peaks at 0.52, 1.4, and 1.87 V and exhibits oxidation peaks at 1.8 and 2.31 V (Fig. S2b). During the reduction reactions, MoS_2_ reacts with lithium, forming MoS_2_, Li_x_MoS_2_, Li_2_S, and Mo, whereas the oxidation reactions reform MoS_2_ (Table S2). This suggests that MoS_2_ can be converted to Li_2_S and Mo when a sufficient amount of Li is supplied. Figure 3d shows the CV curve of the assembled cell based on SE containing MoS_2_-15 m. When an SE is used with the CCs based on MoS_2_-15 m, additional oxidation and reduction reactions can be observed at ~ 2.4 and ~ 2.0 V, respectively. Because SUS does not react with the SE within the specified voltage range [60], these peaks imply that the interface of the SE is converted to PS_4_^3−^ [61] by interaction with the contacted MoS_2_, suggesting that the MoS_2_ in contact with the SE receives a sufficient amount of lithium to initiate the conversion reaction. At the interface between the SE and MoS_2_, the conversion reactions with MoS_2_ can lead to the formation of Li_2_S, Mo metal, and lithium thiophosphate, which can serve as high-quality SEI layers between the CCs and SEs. Moreover, Mo metal improves lithium accommodation and supports uniform deposition [36, 43], allowing for even lithium-metal plating, which is consistent with the SEM results. Meanwhile, in the cross-sectional SEM image of the MoS_2_-15 m-based AFASSBs after charging, the MoS_2_ nanosheet structure was not observed, likely because of the conversion of MoS_2_ to Mo and Li_2_S, which acted as a sacrificial layer and resulted in uniform dispersion, as indicated by the CV results.

The pressure-sensitive paper results, as well as those of SEM and CV, suggest that MoS_2_ can promote uniform lithium deposition; however, these results are insufficient to confirm that the deposited material is Li metal. Thus, to obtain direct evidence of lithium-metal plating and stripping, we carried out XRD analysis to characterize the structural changes and surface states of the CCs. Figure 3e, f shows a comparison of the XRD patterns of the SUS and MoS_2_-15 m samples in their pristine states, after lithium plating, and after lithium stripping. In the pristine state, SUS shows no peaks other than those associated with the substrate, whereas MoS_2_-15 m exhibits additional peaks at 14.1° and 33.1°, corresponding to the (002) and (100) planes of MoS_2_, respectively [52, 62]. After the charging process, an Li metal peak appears near 36° in all samples, indicating the deposition of Li metal that is consistent with the SEM results. Notably, for the MoS_2_-15 m sample, the pure MoS_2_ peaks completely disappear, and peaks associated with Li_2_S and lithium thiophosphate can be observed. This implies that a high-quality SEI layer was formed between the CCs and SEs by the MoS_2_ sacrificial layer, which might have contributed to the improved performance of the AFASSBs [36, 40, 41, 63]. A weak peak associated with lithium thiophosphate is also observed in the SUS sample after charging. Because SUS does not react with SEs or other components in the proposed AFASSB structure [11], this peak may be a by-product of a reaction with the Li metal formed during charging. Such unintended reactions and the resulting SEI layer formation could ultimately lead to unsatisfactory cell performance. Figure 3g–l shows the XPS spectra of Mo 3*d* and S 2*p* from the CC layer of the MoS_2_-15 m-based AFASSBs in the pristine state (Fig. 3g, j), after Li plating (Fig. 3h, k), and after Li stripping (Fig. 3i, l). In the deconvoluted core-level spectrum of Mo 3*d* in the pristine state (Fig. 3g), two characteristic MoS_2_ peaks corresponding to Mo^4+^ 3*d*_3/2_ and Mo^4+^ 3*d*_5/2_ can be identified at 232.1 and 229.0 eV, respectively. In the same spectrum, a peak corresponding to S 2*s* is observed at 226.2 eV. From the S 2*p* core-level spectra of MoS_2_ in the pristine state (Fig. 3j), two peaks corresponding to the S 2*p*_1/2_ and S 2*p*_3/2_ bonding states of MoS_2_ can be identified at 163.3 and 162.1 eV, respectively. The binding energies of the Mo 3*d*, S 2*s*, and S 2*p* peaks in the pristine state display typical properties of MoS_2_, which is consistent with previous reports [46, 64, 65]. In the Mo 3*d* core-level spectrum after lithium plating, as shown in Fig. 3h, the characteristic peaks of 2H MoS_2_ (Mo^4+^ 3*d*_3/2_ and Mo^4+^ 3*d*_5/2_) have disappeared, and new peaks have emerged in the lower-energy region (224–229 eV). The Mo 3*d* spectrum in the 224–229 eV binding energy region was deconvoluted into two peaks by fitting. The blue peak at 227.8 eV and the red peak at 225.7 eV correspond to the Mo 3*d* of Mo metal and the S 2s properties of Li_2_S, respectively, indicating a conversion reaction occurring at the SE/MoS_2_ interface [37]. This conversion reaction is further supported by the appearance of the S 2*p*_1/2_ and S 2*p*_3/2_ peaks of Li_2_S at 161.3 and 160.2 eV, respectively, as shown in Fig. 3k [37]. The presence of Mo metal and Li_2_S produced from the conversion reaction indicates the formation of an interlayer with a lithium-friendly component and a high-quality SEI layer, which may positively affect the performance of AFASSBs [36, 40, 41]. After the lithium stripping process (Fig. 3i, l), the Mo metal and Li_2_S layers remain, suggesting that the interlayer remains stable at the interface throughout the charge–discharge cycles. This robust interlayer contributes to improved cycling stability in both asymmetric and full cells.

### Electrochemical Performance of Anode-Free All-Solid-State Batteries

**Fig. 4:**
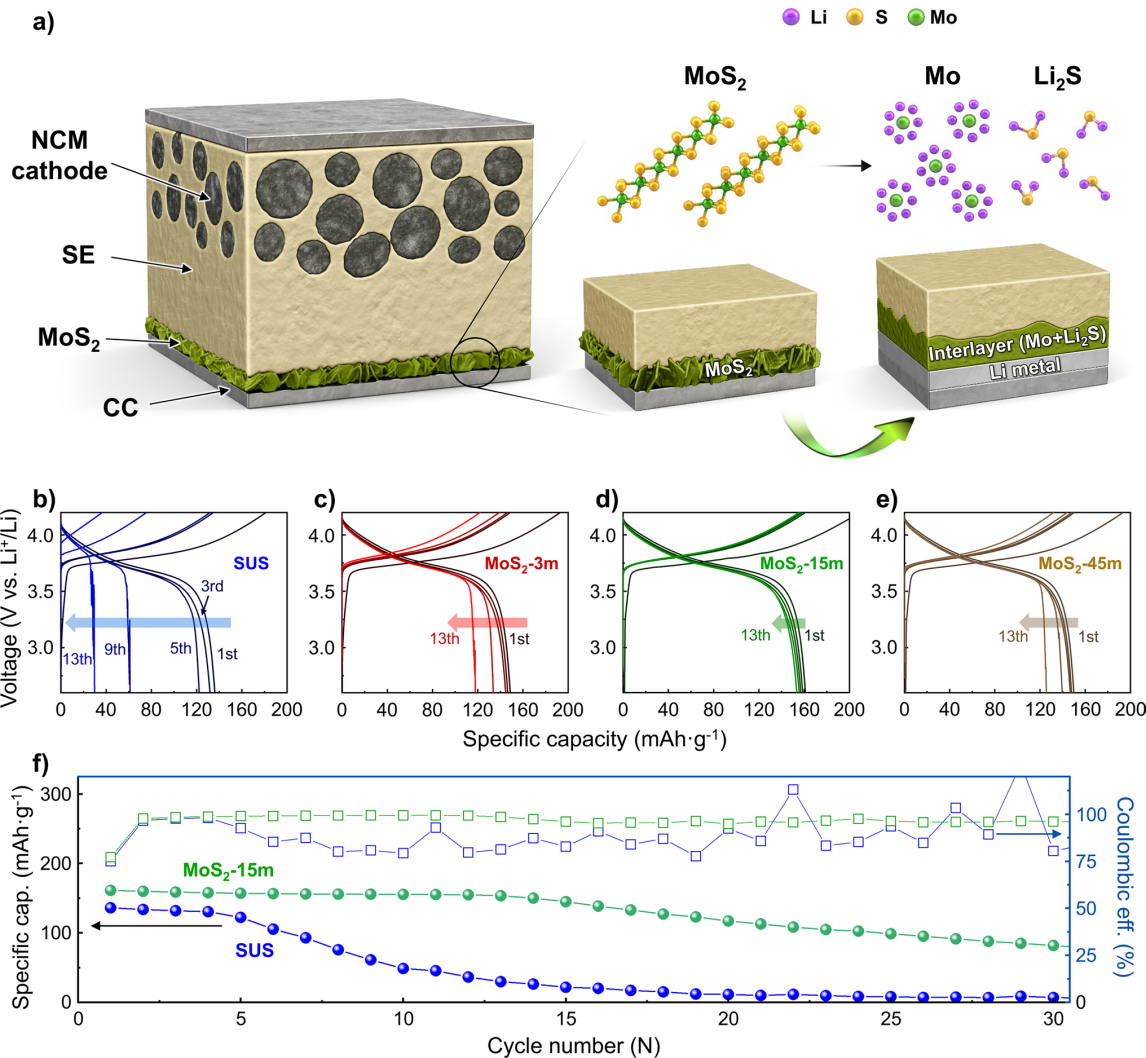
**a** Schematic depiction illustrating the structure and interface formation procedure for Li-free full cells based on NCM cathode. Full-cell evaluation of anode-less electrode with **b** SUS, **c** MoS_2_-3m, **d** MoS_2_-15m, and **e** MoS_2_-45m. **f** Cycling performance of the SUS and MoS_2_-15m at a current density of 0.2C

To further validate the strategy proposed in this study, Li-free full cells based on NCM 622 cathodes were assembled as shown in Fig. 4a, and the performances of AFASSBs with various samples (SUS CCs, MoS_2_-3 m, MoS_2_-15 m, and MoS_2_-45 m) were evaluated. Figure 4b–e presents the galvanostatic charge–discharge curves of each full cell at 0.2C and 60 °C. Figure 4f compares the capacities and coulombic efficiencies across the cycle numbers of the SUS CC- and MoS_2_-coated CC-based AFASSBs. The initial discharge capacities of the SUS, MoS_2_-3 m, MoS_2_-15 m, and MoS_2_-45 m cells were 136.1 mAh g^−1^ (Fig. 4b), 148.7 mAh g^−1^ (Fig. 4c), 161.1 mAh g^−1^ (Fig. 4d), and 149.9 mAh g^−1^ (Fig. 4e), respectively. All MoS_2_-based cells exhibited higher initial discharge capacities than did the SUS cell, likely because of the enhanced Li storage capability of the CC surface owing to the formation of an interlayer containing Mo metal (Fig. 4a) [43]. Despite having the same charge and discharge profiles, the AFASSBs with the SUS CCs experienced severe capacity deterioration before they reached the 10th cycle, as shown in Fig. 4b. This performance deterioration can be attributed to the unstable interface between the SUC CCs and SEs caused by poor Li affinity on the surface. In AFASSBs, the unstable interface during Li deposition and stripping may result in nonuniform Li deposition and localized high current densities, which then lead to Li dendrite growth and the rapid deterioration of cell performance [58]. In contrast, the Li-free full cell with MoS_2_-coated CCs exhibited a significantly improved cycling performance relative to the pristine SUS, regardless of the growth conditions (Fig. 4c–e). This suggests that the interlayer formation with Mo metal and Li_2_S through the conversion reaction of MoS_2_ induces better lithium plating/stripping behavior in the AFASSB system, which is consistent with the asymmetric cell and structural results (Figs. 2 and 3). Comparing the full-cell performance of various MoS_2_ samples after 13 cycles, the discharge capacity of the cells with MoS_2_-3 m, MoS_2_-15 m, and MoS_2_-45 m was determined to be 117.8, 153.4, and 125.6 mAh g^−1^, respectively, corresponding to coulombic efficiencies of 96.54%, 98.57%, and 95.02%, respectively. The AFASSBs containing MoS_2_-3 m exhibited a deterioration in discharge capacity after nine cycles, which could be attributed to the insufficient size of the MoS_2_ nanosheets or the locally exposed, unstable CC surface without MoS_2_. In contrast, the MoS_2_-15 m and MoS_2_-45 m with larger MoS_2_ nanosheets demonstrated better performance and stability in cycling evaluations. Meanwhile, the discharge capacity and capacity retention observed for MoS_2_-45 m were relatively less satisfactory than those of MoS_2_-15 m, which could be attributed to the high lithium consumption due to the excessive size and density of MoS_2_ during the conversion reaction. Furthermore, an excessively thick MoS_2_ layer can lead to a thicker SEI layer at the interface with the SE, increasing overall cell resistance and ultimately degrading cycling performance [66]. This suggests that when the amount of cyclable Li is limited, as in the case of lithium-free full cells, there is an appropriate size and thickness, for the interlayer based on MoS_2_ to serve its role properly. Figure 4f shows the capacities and coulombic efficiencies of SUS CCs and MoS_2_-15 m over multiple cycles. When the size of the MoS_2_ nanosheets is not optimal, unsatisfactory performance or rapid capacity degradation may result, whereas AFASSB systems with optimally sized MoS_2_ nanosheets demonstrate a greater than sevenfold better capacity retention over SUS CCs. As shown in Fig. 4f, the bare SUS CCs without a MoS_2_ layer showed severe capacity fading, with a capacity retention of only 8.3% after 20 cycles (average coulombic efficiency of 86.4%), whereas the AFASSBs with MoS_2_-15 m achieved a capacity retention of 58.9% after 20 cycles with an average coulombic efficiency of 96.7%. To gain further insights into the performance enhancement achieved by incorporating MoS_2_ in the full cell, EIS analysis was performed. The Nyquist plot of bare SE was fitted using a simplified Randles circuit (inset of Fig. S4a), revealing bulk resistance and grain boundary (GB) resistance values of 62.84 Ω and 64.3 Ω, respectively. The Nyquist plots obtained after assembling cells with SUS CC and MoS_2_-15 m were fitted with two semicircles (Fig. S4b, c) [67]. While the bulk and GB resistances of SE remained nearly unchanged, the green semicircle corresponding to MoS_2_-15 m was significantly smaller than that of SUS CC. Since the radius of the semicircle represents charge transfer resistance, a smaller radius indicates improved Li-ion transport properties [68, 69]. Thus, the charge transfer resistance of MoS_2_-15 m was substantially lower than that of SUS CC (Table S3), likely due to the enhanced interfacial contact between SE and CC, facilitated by the vertical MoS_2_ nanosheets. Even after charge cycling, the charge transfer resistance of SUS CC (Fig. S4d) remained more than twice that of MoS_2_-15 m (Fig. S4e) due to poor interfacial contact between SE and CC. This further emphasizes the significant role of interfacial improvements introduced by MoS_2_-15 m in enhancing full-cell performance. Furthermore, the AFASSBs with the MoS_2_-coated CCs demonstrated better cycling properties than the bare SUS CCs, regardless of the synthesis conditions (Fig. S5). This implies that the control of interlayer formation and morphology based on MoS_2_ shows significant synergistic effects in achieving uniform Li deposition and stripping in the AFASSB system.

## Conclusions

This study reports an efficient strategy for improving the interface between the SEs and CCs of AFASSBs using controllable MoS_2_. Vertical MoS_2_ nanosheets were controllably grown on CCs using MOCVD. The MoS_2_ nanosheets in contact with the SEs provided a uniform contact area and served as a sacrificial thin film, forming an intermediate layer of Mo metal and Li_2_S via conversion reactions. Systematic structural and electrochemical analyses of the samples demonstrated that the addition of MoS_2_ to the AFASSBs decreased the nucleation overpotential of Li and enabled uniform Li formation at the interface owing to the formation of Li_2_S and Mo metal. Furthermore, the AFASSB full cell fabricated with NCM cathodes operated successfully, demonstrating enhanced cycling stability compared with bare cells, with the cell properties influenced by the morphology and size of MoS_2_. Consequently, the AFASSBs with MoS_2_-15 m, with optimally sized nanosheets, showed significantly better capacity retention of 58.9% relative to the 8.3% for bare SUS. These results suggest a facile and promising approach for exploiting the potential of MoS_2_ for efficient AFASSB applications.

## Supplementary Information

Below is the link to the electronic supplementary material.

Supplementary file1 (DOCX 422 KB)
