# Supplementary material for: Tailoring Artificial Solid Electrolyte Interphase via MoS2 Sacrificial Thin Film for Li-Free All-Solid-State Batteries
Source: Nanomicro Lett. 2025 Apr 18;17:224. doi: 10.1007/s40820-025-01729-w (PMC12008103; doi:10.1007/s40820-025-01729-w)
Supplement: Supplementary file 1 — Supplementary file1 (DOCX 422 KB) [file 40820_2025_1729_MOESM1_ESM.docx]

Supporting Information for

**Tailoring Artificial Solid Electrolyte Interphase via MoS_2_ Sacrificial Thin-Film for Li-Free All-Solid-State Batteries**

Dong-Bum Seo^1,†^, Dohun Kim^2,†^, Mee-Ree Kim^2^, Jimin Kwon^2^, Hyeong Jun Kook^3^, Saewon Kang^1^, Soonmin Yim^1^, Sun Sook Lee^1^, Dong Ok Shin^3^, Ki-Seok An^1,^*, and Sangbaek Park^2,^*

^1^Thin Film Materials Research Center, Korea Research Institute of Chemical Technology (KRICT), 141 Gajeong-ro, Yuseong-gu, Daejeon 34114, Republic of Korea

^2^Department of Materials Science and Engineering, Chungnam National University, Daejeon, 34134, Republic of Korea

^3^Intelligent Sensors Research Section, Electronics and Telecommunications Research Institute (ETRI), Daejeon, 34129, Republic of Korea

† Dong-Bum Seo and Dohun Kim contributed equally to this work.

* Corresponding authors. E-mail: [ksan@krict.re.kr](mailto:ksan@krict.re.kr) (Ki-Seok An); [sb.park@cnu.ac.kr](mailto:sb.park@cnu.ac.kr) (Sangbaek Park)

Supplementary Figures and Tables


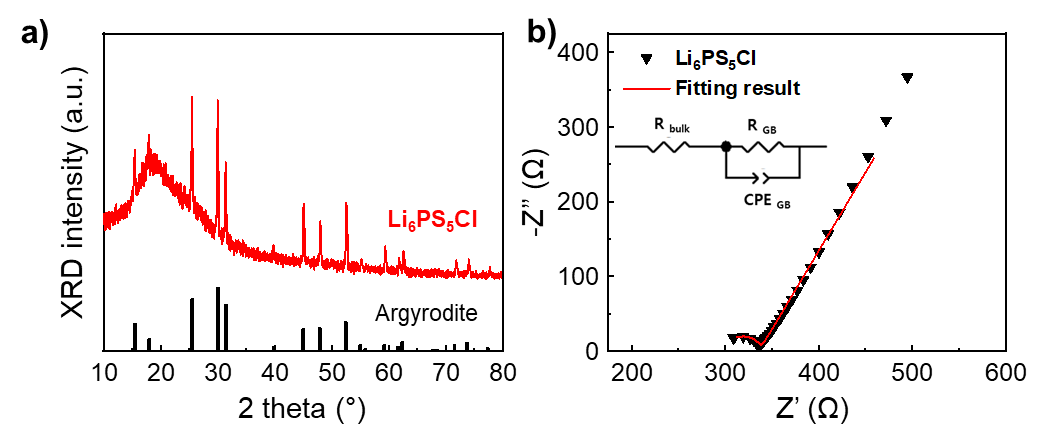


**Fig. S1** (**a**) XRD pattern of prepared Li_6_PS_5_Cl electrolyte. (**b**) Nyquist plots of Li_6_PS_5_Cl at RT

**Table S1** Electrochemical impedance spectroscopy (EIS) properties of Li_6_PS_5_Cl as calculated from the Nyquist plot

| R_bulk_ (Ω) | R_GB_ (Ω) | Impedance (Ω) | Area (cm^2^) | Thickness (cm) | Conductivity (mS cm^-1^) | |
| --- | --- | --- | --- | --- | --- | --- |
| 299.31 | 37.14 | 336.44 | 0.2826 | 0.171 | | 1.8 |


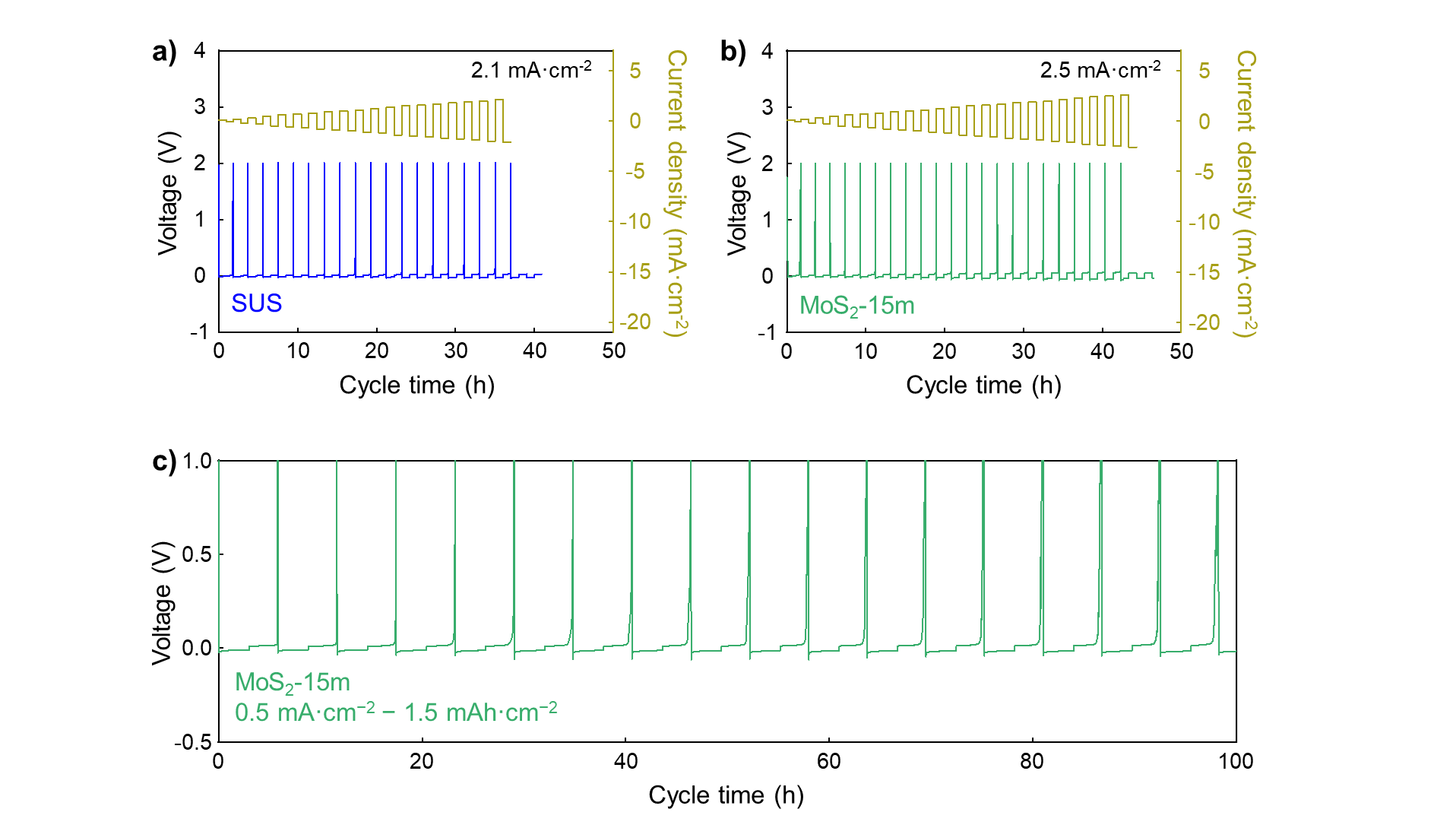


**Fig. S2** The critical current density (CCD) analysis of (**a**) SUS and (**b**) MoS_2_-15m. (**c**) The galvanostatic cycling test of MoS_2_-15m conducted at a current density of 0.5 mA cm^−2^ with an areal capacity of 1.5 mAh cm^−2^


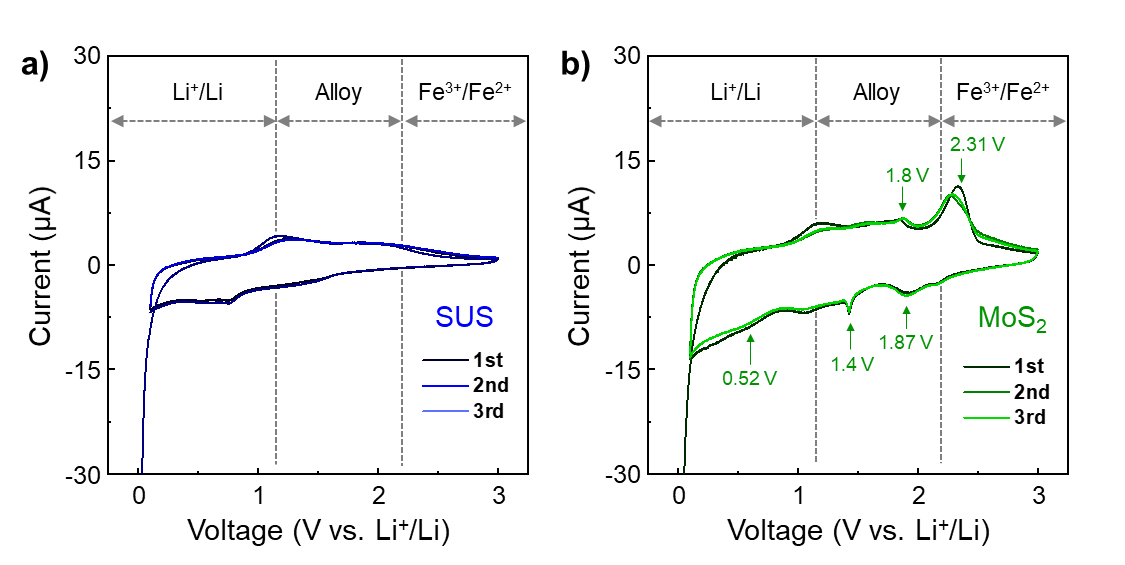


**Fig. S3** CV curve obtained using a liquid electrolyte with (**a**) SUS and (**b**) MoS_2_-15m

**Table 2** Redox reaction voltage value and equation with MoS_2_ based on liquid electrolyte

| **Voltage (V)** | **Redox reaction equation** |
| --- | --- |
| 0.52 | Li_x_MoS_2_ + 4Li+ + 4e^-^ → 2Li_2_S + Mo |
| 1.4 | MoS_2_ + 4Li+ + 4e^-^ → Li_x_MoS_2_ |
| 1.87 | 2Li^+^ + 2e^-^ + S → Li_2_S |
| 1.8 | Mo + 2S → MoS_2_ |
| 2.31 | Li_2_S → 2Li+ + 2e^-^ + S |


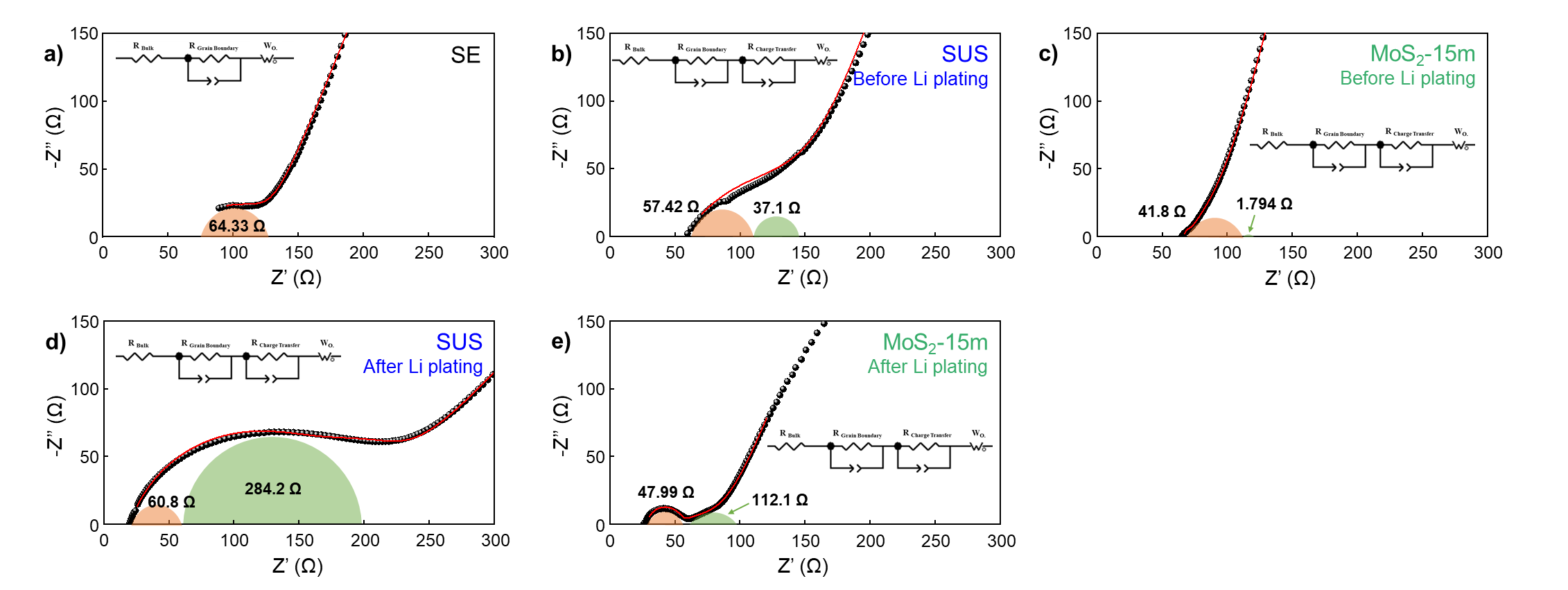


**Fig. S4** (a) Nyquist plots of Li_6_PS_5_Cl. (**b-c**) Nyquist plots of full-cell structures with (**b**) SUS and (**c**) MoS_2_-15m before Li plating. (**d-e**) Nyquist plots of full-cell structures with (**d**) SUS and (**e**) MoS_2_-15m after Li plating

**Table 3** EIS properties of SUS CC and MoS_2_-15m as calculated from the Nyquist plot

| **Structure** | **Lithium Plating** | **R _Bulk_**  **(Ω)** | **R _Grain Boundary_**  **(Ω)** | **R _Charge Transfer_**  **(Ω)** |
| --- | --- | --- | --- | --- |
| SUS \| LPSCl \| SUS | - | 62.84 | 64.3 | - |
| Cathode \| LPSCl \| SUS | Before | 57 | 57.42 | 37.1 |
| Cathode \| LPSCl \| SUS | After | 39.88 | 60.8 | 284.2 |
| Cathode \| LPSCl \| MoS_2_ | Before | 65.72 | 41.8 | 1.794 |
| Cathode \| LPSCl \| MoS_2_ | After | 54.51 | 47.99 | 112.1 |


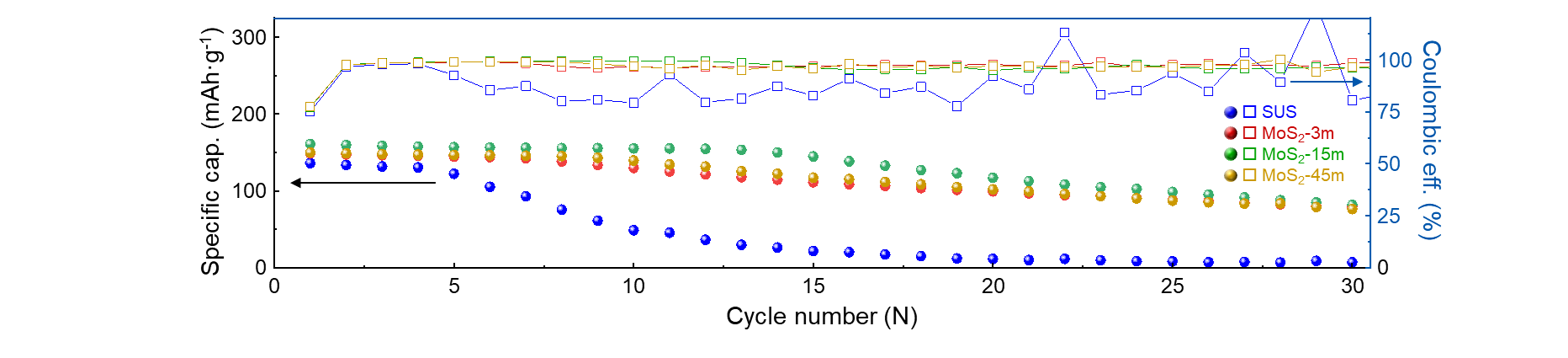


**Fig. S5** Cycling performance of various samples (SUS, MoS_2_-3m, MoS_2_-15m, and MoS_2_-45m) at a current density of 0.2 C
